# Knowledge about the intrauterine device and interest in using it among women users of primary care services*

**DOI:** 10.1590/1518-8345.3140.3232

**Published:** 2020-02-14

**Authors:** Ana Luiza Vilela Borges, Karina Simão Araújo, Osmara Alves dos Santos, Renata Ferreira Sena Gonçalves, Elizabeth Fujimori, Eveline do Amor Divino

**Affiliations:** 1 Universidade de São Paulo, Escola de Enfermagem, São Paulo, SP, Brazil.; 2 Hospital Municipal Universitário de São Bernardo do Campo, São Bernardo do Campo, SP, Brazil.; 3 Centro Universitário das Faculdades Metropolitanas Unidas, São Paulo, SP, Brazil.; 4 Universidade Federal de Mato Grosso, Faculdade de Enfermagem, Cuiabá, MT, Brazil.

**Keywords:** Contraception, Intrauterine Devices, Sexual and Reproductive Health, Women’s Health, Primary Health Care, Nursing, Anticoncepção, Dispositivos Intrauterinos, Saúde Sexual e Reprodutiva, Saúde da Mulher, Atenção Primária à Saúde, Enfermagem, Anticoncepción, Dispositivos Intrauterinos, Salud Sexual y Reproductiva, Salud de la Mujer, Atención Primaria de Salud, Enfermería

## Abstract

**Objective:**

to analyze the level of knowledge about the intrauterine device, the interest in using it and the relationship between these events among women in reproductive age.

**Method:**

cross-sectional study conducted with 1858 women between 18 and 49 years old, attending Primary Health Care Facilities. Data were obtained in face-to-face interviews. The level of knowledge was evaluated by items with answers options “agree”, “disagree” and “I don’t know”. Knowledge was categorized as below/equal and above the median. Chi-square and multiple logistic regression were used in Stata 14.2 (95% confidence level).

**Results:**

intrauterine device current use was not frequent (1.7%; n=32) and the level of knowledge was higher among women between 25 and 34 years old, white, living in Aracaju (Sergipe), who were more educated, and who were currently using or had already used the intrauterine device. Interest in using the intrauterine device (38.0%; n=634) was higher among younger women, single, more educated, had health insurance, no children and with higher level of knowledge about the intrauterine device.

**Conclusion:**

the level of knowledge about the intrauterine device was associated with the interest in using it.

## Abstract (pt)

**Objetivo:**

analisar o nível de conhecimento sobre o dispositivo intrauterino, o interesse em usá-lo e a relação desses eventos entre mulheres em idade reprodutiva.

**Método:**

estudo transversal conduzido com 1858 mulheres de 18-49 anos de idade, usuárias de Unidades Básicas de Saúde. Os dados foram obtidos por entrevistas face a face. Avaliou-se o nível de conhecimento por meio de itens que admitiam repostas do tipo concordo, não concordo e não sei, cujo escore foi categorizado em abaixo/igual e acima da mediana. Utilizaram-se qui-quadrado e regressão logística múltipla, calculados no Stata 14.2 (nível de confiança igual a 95%).

**Resultados:**

o uso atual do dispositivo intrauterino foi pouco frequente (1,7%; n=32), sendo o nível de conhecimento maior entre mulheres com 25-34 anos de idade, mais escolarizadas, brancas, que usavam/já tinham usado o dispositivo intrauterino e residentes em Aracaju, Sergipe. O interesse em usar o dispositivo intrauterino (38,0%; n=634) foi maior entre as mulheres mais jovens, com maior escolaridade, com plano de saúde, solteiras, sem filhos e com maior nível de conhecimento sobre o dispositivo intrauterino.

**Conclusão:**

o nível de conhecimento sobre o dispositivo intrauterino foi associado ao interesse em usá-lo.

## Abstract (es)

**Objetivo:**

analizar el nivel de conocimiento sobre el dispositivo intrauterino, el interés en usarlo y la relación de estos eventos entre las mujeres en edad reproductiva.

**Método:**

estudio transversal realizado con 1858 mujeres entre 18 y 49 años, usuarias de Unidades Básicas de Salud. Los datos se obtuvieron a través de entrevistas personales. El nivel de conocimiento se evaluó mediante ítems que permitieron respuestas del tipo de acuerdo, en desacuerdo y no sé, cuyo puntaje se clasificó como inferior/igual y superior a la mediana. Se utilizaron chi-cuadrado y regresión logística múltiple, calculados en el Stata 14.2 (nivel de confianza del 95%).

**Resultados:**

el uso actual del dispositivo intrauterino fue poco frecuente (1,7%; n=32) y el nivel de conocimiento fue mayor entre las mujeres blancas de 25 a 34 años, más educadas, que ya usaban o habían usado el dispositivo intrauterino y residentes en Aracaju, Sergipe. El interés en usar el dispositivo intrauterino (38,0%; n=634) fue mayor entre las mujeres más jóvenes, con mayor nivel educativo, con seguro médico, solteras, sin hijos y con un mayor nivel de conocimiento sobre el dispositivo intrauterino.

**Conclusión:**

el nivel de conocimiento sobre el dispositivo intrauterino se asoció con el interés en usarlo.

## Introduction

The intrauterine device (IUD) is a contraceptive method in the group of long-acting reversible contraception, or LARC, and it is highly effective and safe^(^ 1 ^)^. Although it is the most widely used reversible method in the world^(^ 2 ^)^, it is still underused in North America, South Asia, Oceania and sub-Saharan Africa^(^ 3 ^)^, as well as in Brazil.

In Brazil, data from the third and most recent edition of the Demographic and Health Survey (DHS), conducted in 2006, revealed that the IUD was used by 1.5% of women^(^ 4 ^)^, despite of the fact that the copper IUD is available in the Unified Health System (SUS); the levonorgestrel intrauterine system is not available in SUS. Reasons for its underutilization include insufficient and discontinuous provision of the method, lack of health professionals qualified for its insertion, unnecessary and excessive eligible criteria established in certain services, inadequate knowledge of the method among health professionals, and the poor awareness of women and couples about its mechanism of action, safety and effectiveness, among others^(^ 5 ^-^ 7 ^)^.

Specifically about IUD knowledge, it is a method surrounded by misconceptions among women^(^ 8 ^-^ 12 ^)^. In this sense, many think that it can cause infertility or cancer, that is not indicated either for young or nulliparous women, and others express great fear about the insertion procedure^(^ 8 ^-^ 12 ^)^. Perceptions and fears such as these are common in many contexts; however, studies that address the issue suggest that if health services and professionals provide timely and adequate information about the method, confidence in the IUD can be increased, consequently the motivation to use it may increase^(^ 13 ^)^.

Increasing IUD participation in the contraceptive method mix is a good strategy to reduce the occurrence of unintended pregnancies^(^ 14 ^)^, which reach 55% in Brazil^(^ 15 ^)^, and, consequently, decrease the number of unsafe abortions. In addition to being one of the most effective reversible methods, IUD is also suitable for groups that may have difficulty with continuous methods, such as young women or women with sporadic sexual behavior, and it also may be used in the postpartum and post abortion periods^(^ 16 ^)^. IUD users are among the most satisfied women using contraceptive methods^(^ 17 ^)^, and satisfaction with contraception is associated with high rates of use continuation^(^ 18 ^)^. 

Thus, investigating the knowledge about the IUD and the interest in using it can provide insights to explain the low percentage of use in the country and to find strategies to promote and expand its use. A review of studies on the knowledge of health professionals and of women/couples about the IUD emphasized that in low- and middle-income countries, such as Brazil and other Latin American countries, there is a lack of information on the perspective of these groups regarding IUDs, which in turn, limits the knowledge about what women consider when choosing or rejecting a contraceptive^(^ 19 ^)^. The objective of this study was to analyze the level of knowledge on the IUD, the interest in using it and the relationship between these events among women in reproductive age who were users of Primary Health Care Facilities (PHCF) in the three cities in Brazil. 

## Method

We conducted a quantitative, cross-sectional study with women ranging 18 to 49 years of age, who were users of PHCF from three Brazilian cities: São Paulo (São Paulo state), Aracaju (Sergipe state) and Cuiabá (Mato Grosso state).

The parameters to determine the sample size in each city were: proportion of women estimated equal to 50%; 95% confidence level; relative sampling error of 5%; and design effect (deff) of 2^(^ 20 ^)^. Sample size calculation indicated the need for 768 women, 800 in São Paulo and 385 in Aracaju and 368 in Cuiabá. Considering that the estimates should be obtained from women using contraceptive methods, which have an estimated proportion of 80%^(^ 4 ^)^, the number of women who should be interviewed was 1000 in São Paulo and 482 in Aracaju and in Cuiabá. Then, considering the percentage of 25% of women who would not answer the questionnaire (refusal or loss due to interviewer’s problems) and 33% of women between 18 and 49 years old who would not be eligible for the interview (the exclusion criteria were never having had sexual intercourse, using a permanent contraceptive method and reporting not being aware of IUD)^(^ 4 ^)^. Thus, 1993 women should be selected in the city of São Paulo, in order to obtain 1000 valid interviews, and 963 women in Aracaju and 963 in Cuiabá, in order to obtain at least 482 valid interviews in each of these capitals. 

The sampling plan was conducted in two-stage cluster sampling. The first-stage (primary sampling unit) consisted of the PHCF, drawn with probability proportional to size, measured by the number of cytopathological exams performed in 2014. Based on this criterion, 38 PHCF were selected in São Paulo, out of 441; 19 PHCF in Aracaju out of 43; and 19 in Cuiabá out of 93. In the three capitals, the interviews were conducted in three consecutive days in each PHCF and, on each day, nine women were interviewed, or 27 valid interviews per day per PHCF. Selected PHCF are geographically distributed throughout the different regions of these cities, including the central region and extremes on the periphery. 

In the second stage of selection, women to be interviewed in each PHCF were not randomly selected, according to the following criteria: 1) women waiting for pap smear test; 2) women waiting for medical or nursing consultation; 3) women waiting for any other type of intervention. It was not possible to randomly select women, because there are several forms of organizing to collect this exam at the PHCF, such as scheduling all women at the same time or according to demand, without any kind of scheduling. In addition, not every PHCF offered pap smear test that moment, so women were referred to another PHCF. Altogether, 3317 women were invited to participate in the study; 225 refused to participate and 1022 fit the exclusion criteria (not having started sexual life and using a permanent contraceptive method). Thus, 2070 women were interviewed.

Data was collected in face-to-face interviews conducted by health professional researchers (nurses, psychologists and midwives). Women were approached by the interviewers and invited to participate in the research. The objectives, the content of the questions and the stages of the interview were explained. Women who agreed to participate in the research signed the Informed Consent Form. The interviews were conducted at the PHCF through a structured instrument, using the platform Census and Survey Processing System - CSPro in tablets. Interviews occurred from October to December 2015 in São Paulo/SP, from August to September 2016 in Aracaju/SE, and from August to October 2017 in Cuiabá/MT. The instrument was developed by the researchers and was pre-tested with 17 women in two PHCF in the city of São Paulo, not selected for this study. The instrument contained questions about socio-demographic characteristics (e.g. age, race/color, education, work, possession of material goods, union status, among others), reproductive history (e.g. age at menarche, age at the beginning of sexual life, number of previous pregnancies, use of contraceptive methods in the last sexual intercourse), knowledge about the IUD, use of IUD (previous and current use) and desire to use the IUD. 

In order to elaborate the variable “level of knowledge about the IUD”, 12 statements about the mechanisms of action, indications for use and side effects of the IUD were elaborated, based on studies that also measured the level of knowledge about the IUD^(^ 10 ^-^ 11 ^,^ 21 ^-^ 24 ^)^.

Women who knew or had heard about the IUD responded to each statement with the following response options: “agree”, “disagree” or “I don’t know”. Because knowledge is a latent variable, that is, one that cannot be directly measured or observed, its analysis considered the subjectivity and the difficulty of “measuring” women’s knowledge about the IUD^(^ 25 ^)^. This was done through three strategies: 1) Item Response Theory (IRT); 2) Factor analysis, with an eigenvalue greater than one, according to the Kaiser criterion; 3) Cronbach’s Alpha. The intention was to choose, based on these strategies, which statements should compose the construct “knowledge level about the IUD”. 

The IRT showed that the following items presented moderate, high or very high discrimination and therefore were considered in the next step (factor analysis): item 4 - The man feels the IUD during sexual intercourse (correct option=disagree, discrimination index 1.59); item 7 - The IUD is inserted through surgery (correct option=disagree, discrimination index 1.94); item 8 - The IUD is abortive (correct option=disagree, discrimination index 1.58); item 9 - After removing the IUD, women have difficulty getting pregnant (correct option=disagree, discrimination index 1.80); item 10 - The IUD increases the risk of uterine cancer (correct option=disagree, discrimination index 1.99); and item 12 - The IUD has many unpleasant side effects (correct option=disagree, discrimination index 1.45).

The factor analysis confirmed that these six items were part of a single factor (Eigenvalue equal to 1.665), that is, they compound a single latent construct/variable, which is IUD knowledge. Also, all items showed factor loadings greater than 0.30 (item 4=0.540; item 7=0.390; item 8=0.541; item 9=0.571; item 10=0.597; item 12=0.494). Kaiser-Meyer-Olkin (KMO) was 0.798, which shows that the data set was adequate to proceed with factor analysis^(^ 26 ^)^. Finally, Cronbach’s alpha coefficient was 0.706, indicating that this latent variable had adequate reliability. 

Correct answers were coded as 1, while incorrect and “I don’t know” answers were coded as zero. Each woman had her score added up, obtaining a final score from zero to 6. Thus, the higher the score, the higher the level of knowledge. To characterize women according to the level of knowledge about the IUD, the analysis compared those with scores below or equal to the median (≤3), and above the median (>3). The aspects associated with the level of IUD knowledge were analyzed using the chi-square test and, subsequently, with multiple logistic regression, with simultaneous entry of the independent variables, which were the socio-demographic and reproductive characteristics. 

The independent variables analyzed were: age (18-24, 25-29, 30-34 and 35 years or more); race/skin color (white, brown, black and yellow/indigenous); level of education (up to 8 years, 9 to 11 years and 12 years or more of education); paid job (no/yes); health insurance (no/yes); stable relationship, that is, living with a partner (no/yes); current pregnancy (no/yes); number of children (none, one, two, three and more); previous abortion (no/yes); reproductive intention (wants [more] children, does not want [more] children, and does not know); and contraceptive method use (none, irreversible, hormonal, LARC, barrier and traditional). In the logistic regression analysis, the variable use of contraceptive methods also considered women who were pregnant, so it had one more category, “is currently pregnant”. The main covariate was IUD use (previous and/or current).

For the analysis of interest in using the IUD, women with no female sterilization and with partners without vasectomy, who did not use IUD and had never used it, were asked the reasons why they had never used it and if they would like to use it in the future. Women who did not know if they would like to use IUD or who said that they would not want to use it expressed their reasons. Aspects associated with future interest in using an IUD were analyzed using the chi-square test. Subsequently, multiple logistic regression was conducted, with simultaneous entry of the independent variables already mentioned. In this model, the main covariate was the level of knowledge on the IUD. The dependent variables were the level of knowledge on the IUD (dichotomous: below the median and equal to or greater than the median) and the interest in using IUD (also dichotomous: no and yes). All data were analyzed in Stata 14.2. A confidence level of 95% was considered and the Hosmer-Lemeshow test was applied to check models goodness of fit.

The project was approved by the local Research Ethics Committee and the Research Ethics Committee of the Municipal Health Office of São Paulo (protocol 1.876.178/2016). The authorizations of the health offices of the cities of São Paulo/SP, Aracaju/SE and Cuiabá/MT were obtained before beginning data collection.

## Results

As the purpose of this study was to analyze the relationship between knowledge on the IUD and interest in using it, the sample consisted of 1858 women who were aware of the method: 957 from São Paulo/SP, 441 from Aracaju/SE and 460 from Cuiabá/MT. 

Women had a mean age of 30.5 years (SD=7.8) and their partners had a mean age of 33.7 years (SD=8.9). Mean age at menarche was 12.6 years (SD=1.8 first); mean age at first sexual intercourse was 17.0 years (SD=3.1); and mean age of first pregnancy was 20.9 years (SD=5.1). 

Most women were under 35 years old and declared themselves as brown (52.0%); 15.7% continued to study after finishing high school and 17.6% had health insurance. Most worked, lived with a partner and had one or more children. Almost a quarter (23.9%) was pregnant at the time of the interview. Previous abortion was reported by 23.3% of the women. Among the participants, 60.2% answered they did not want to have (more) children; 80% of non-pregnant women used contraception; almost half used hormonal methods (49.3%) and 2.4% used LARC. Thirty-two women were using an IUD at the time of the interview (1.7%). Another 67 women reported that they had used an IUD before, totaling 99 women with previous/current IUD use. Socio-demographic and reproductive characteristics differed among women depending on the city they lived and in relation to age, skin color, education, health insurance, civil status, current pregnancy and previous abortions. For example, women residing in Cuiabá/MT were younger, brown and had a higher level of education compared to those from São Paulo/SP and Aracaju/SE. In turn, women living in Aracaju/SE reported more experiences of abortions (Table 1).

**Table 1:** Socio-demographic and reproductive characteristics of women users of Basic Health Units. São Paulo, SP, Aracaju, SE and Cuiabá, MT, Brasil, 2015-2017

Variables	Total	
	n	%
Age (years)		
18-24	496	26.7
25-29	425	22.9
30-34	370	19.9
35 or more	567	30.5
Race/skin color		
White	469	25.3
Brown	964	52.0
Black	323	17.4
Asian/indigenous	99	5.3
Level of education (years)		
Up to 8	468	25.2
9-11	1099	59.1
12 or more	291	15.7
Paid job		
No	867	46.7
Yes	991	53.3
Health insurance		
No	1530	82.3
Yes	328	17.6
Stable relationship		
No	479	25.8
Yes	1379	74.2
Number of children		
None	407	21.9
One	672	36.2
Two	455	24.5
Three or more	324	17.4
Previous abortion*		
No	1426	76.7
Yes	432	23.3
Reproductive intention		
Wants (more) children	623	33.5
Does not want (more) children	1118	60.2
Does not know	117	6.3
Contraceptive method use^†^		
None	305	21.6
Irreversible	99	7.0
Hormonal	697	49.3
IUD^‡^ and implant	34	2.4
Barrier	238	16.8
Traditional	41	2.9
Total	1858	100

* Previous abortion = Includes women who had never been pregnant;

† Use of contraceptive method = Does not consider women who were pregnant at the moment of the interview;

‡ IUD = Intrauterine device

Table 2 shows the 6 items that made up the latent variable “knowledge on the IUD”. With the exception of two items, in all others, less than half of the women answered correctly. The median of correct answers was 3; 7.2% of women got all six items right (n=134) and 15.3% got all of them wrong (n=284). 

**Table 2:** Number and proportion of women users of Basic Health Units, according to knowledge about the IUD*. São Paulo, SP, Aracaju, SE and Cuiabá, MT, Brasil, 2015-2017

Statements	Agrees		Does not agree		Does not know	
	n	%	n	%	n	%
The IUD* is abortive^†^	255	13.7	1002	54.0	600	32.3
After IUD removal*, women have difficulty getting pregnant^†^	247	13.3	983	52.9	627	33.8
The IUD* is inserted through surgery	450	24.2	924	49.7	484	26.1
The man feels the IUD* during sexual intercourse	92	4.9	768	41.3	998	53.7
The IUD* increases the risk of uterine cancer^†^	391	21.0	698	37.6	769	41.4
The IUD* has many unpleasant side effects	581	31.3	538	28.9	739	39.8

* IUD = Intrauterine device;

† Statement = One woman did not answer

Table 3 shows the distribution of the level of knowledge on the IUD, according to socio-demographic and reproductive characteristics of women. In the bivariate analysis, this variable was associated with age, race/skin color, level of education, health insurance, number of children and current and/or previous use of IUD. The multiple logistic regression analysis showed that women between 30 and 34 years old, with a higher level of education and who were using or had used an IUD were more likely to have a higher level of knowledge on the IUD, as well as were women from Aracaju. On the other hand, non-white women, such as brown, black, Asian and indigenous, showed a lower level of knowledge on the IUD compared to white women.

**Table 3:** Socio-demographic and reproductive characteristics of women users of Basic Health Units, according to the level of knowledge about the IUD*. São Paulo, SP, Aracaju, SE and Cuiabá, MT, Brasil, 2015-2017

Variables	Level of knowledge about the IUD* ^†^						
	≤ median		> median		p	ORa^‡^	95% CI^§^
	n	%	n	%			
City							
São Paulo	479	51.7	478	51.3	0.945	1.00	-
Aracaju	217	24.9	224	24.1		1.30	1.01-1.66
Cuiabá	231	23.4	229	24.6		1.02	0.80-1.31
Age (years)							
18-24	282	30.4	214	23.0	<0.001	1.00	-
25-29	213	23.0	212	22.8		1.17	0.89-1.55
30-34	155	16.7	215	23.1		1.59	1.18-2.15
35 or more	277	29.9	290	31.1		1.20	0.90-1.61
Race/skin color							
White	193	20.8	276	29.7	<0.001	1.00	-
Brown	506	54.6	458	49.3		0.67	0.53-0.86
Black	172	18.6	151	16.3		0.63	0.46-0.85
Asian/indigenous	55	5.9	44	4.7		0.59	0.37-0.93
Level of education (years)							
Up to 8	298	32.1	170	18.3	<0.001	1.00	-
9-11	552	59.6	547	58.7		1.88	1.48-2.40
12 or more	77	8.3	214	23.0		4.84	3.38-6.91
Paid job							
No	450	48.5	417	44.8	0.105	1.00	-
Yes	477	51.5	514	55.2		0.99	0.81-1.21
Health insurance							
No	791	85.3	739	79.4	0.001	1.00	-
Yes	136	14.7	192	20.6		1.26	0.96-1.64
Stable relationship							
No	235	25.3	244	26.2	0.673	1.00	-
Yes	692	74.7	687	73.8		1.00	0.80-1.26
Number of children							
None	194	20.9	213	22.9	0.005	1.00	-
One	354	38.2	318	34.1		0.88	0.67-1.16
Two	200	21.6	255	27.4		1.34	0.96-1.87
Three or more	179	19.3	145	15.6		0.99	0.68-1.45
Previous abortion							
No	722	77.9	704	75.6	0.247	1.00	-
Yes	205	22.1	227	24.4		1.11	0.88-1.41
Reproductive intention							
Wants (more) children	312	33.7	311	33.4	0.983	0.94	0.74-1.21
Does not want (more) children	556	60.0	562	60.4		1.00	-
Does not know	59	6.3	58	6.2		0.89	0.58-1.35
Current or previous use of IUD*							
No	911	98.3	848	91.1	<0.001	1.00	-
Yes	16	1.7	83	8.9		4.92	1.84-13.16
Total	927	49.9	931	50.1			

* IUD = Intrauterine device;

† Hosmer-Lemeshow Test (p=0.2376);

‡ ORa = Adjusted odds ratio;

§ CI = Confidence interval

Women who had never used the IUD (n=1759) were asked if they would like to use it, and 58.7% said they would not. The most cited reasons for never having used the IUD was that they were not interested in the method because they were satisfied with their current method (42.1%), followed by the fact that they had no information about the IUD and that it has never been offered to them (26.7%). The most cited reasons for not having interest in using the IUD in the future were lack of interest in the method and satisfaction with the current method (25.1%), followed by fear of the insertion procedure (13.4%) and desire for an irreversible method (12.3%).

Table 4 shows the socio-demographic and reproductive characteristics of women, according to their interest in using the IUD (38.0%). In the bivariate analysis, this variable was associated with: city, age, level of education, health insurance, number of children and level of knowledge on the IUD. Multiple logistic regression analysis showed that the older the woman, the less likely she was to want to use the IUD, compared to women between 18 and 24 years old. In addition, women with 12 years of education or more, single, with health insurance, with children and with a higher level of knowledge on the IUD expressed the most interest in using it. 

**Table 4:** Socio-demographic and reproductive characteristics of women users of Basic Health Units, according to their interest in using the IUD*. São Paulo, SP, Aracaju, SE and Cuiabá, MT, Brasil, 2015-2017

Variables	Interest in using IUD* ^†^						
	No		Yes		P	ORa^‡^	95%CI^§^
	n	%	n	%			
City							
São Paulo	533	51.6	318	50.2	<0.001	1.00	-
Aracaju	274	26.5	125	19.7		0.82	0.62-1.07
Cuiabá	227	21.9	191	30.1		1.21	0.93-1.59
Age (years)							
18-24	253	24.5	234	36.9	<0.001	1.00	-
25-29	221	21.4	173	27.3		0.73	0.54-0.97
30-34	205	19.8	109	17.2		0.45	0.32-0.62
35 or more	355	34.3	118	18.6		0.27	0.19-0.38
Race/skin color^||^							
White	279	27.0	151	23.9	0.085	1.00	-
Brown	521	50.4	335	52.9		1.17	0.89-1.53
Black	166	16.1	119	18.8		1.32	0.94-1.85
Asian/indigenous	67	6.5	28	4.4		0.74	0.44-1.24
Level of education (years)							
Up to 8	284	27.5	126	19.9	0.001	1.00	-
9-11	602	58.2	392	61.8		1.23	0.93-1.61
12 or more	148	14.3	116	18.3		1.48	1.01-2.18
Paid job							
No	479	46.3	293	46.2	0.965	1.00	-
Yes	555	53.7	341	53.8		1.12	0.89-1.39
Health insurance							
No	874	84.5	495	78.1	0.001	1.00	-
Yes	160	15.5	139	21.9		1.38	1.04-1.83
Stable relationship							
No	262	25.3	182	28.7	0.131	1.00	-
Yes	772	74.7	452	71.3		0.67	0.52-0.86
Number of children							
None	262	25.3	142	22.4	0.031	1.00	-
One	380	36.7	262	41.3		1.78	1.32-2.42
Two	223	21.6	152	34.0		2.20	1.50-3.23
Three or more	169	16.3	78	12.3		1.67	1.07-2.59
Previous abortion							
No	810	78.3	493	77.8	0.782	1.00	-
Yes	224	21.7	141	22.2		1.25	0.97-1.62
Reproductive intention							
Wants (more) children	595	57.5	355	56.0	0.192	0.79	0.60-1.04
Does not want (more) children	378	36.6	227	35.8		1.00	-
Does not know	61	5.9	52	8.2		1.20	0.77-1.86
Knowledge on the IUD*							
Below median	577	55.8	285	44.9	<0.001	1.00	-
Above or equal to median	457	44.2	349	55.1		1.60	1.29-1.99
Total	1034	62.0	634	38.0			

* IUD = Intrauterine device;

† Hosmer-Lemeshow Test (p=0.2595);

‡ ORa = Adjusted odds ratio;

§ CI = Confidence Interval;

|| Race/skin color = Two women refused to answer

## Discussion

Our study on the knowledge on the IUD and interest in using it among women attending PHCF in three Brazilian state capitals showed that a reasonable proportion of women had a level of knowledge below the median, that a little more than third were interested in using an IUD in the future and, finally, that the level of knowledge on the IUD was associated with current and previous use of IUD and with the interest in using it.

These results are consistent with findings from other contexts, in which the level of knowledge on the IUD is considered unsatisfactory^(^ 10 ^-^ 12 ^,^ 21 ^,^ 23 ^)^ and is related to socio-demographic variables such as age, education and race/skin color^(^ 27 ^)^. Thus, younger women, self-identified as non-white and with lower level of education had the lowest level of knowledge on the IUD. Studies on reproductive health analyzing the use of contraceptive methods, preconception preparation, pregnancy planning and prenatal care found that groups with this profile face more difficulties to access this type of care and to engage in these practices^(^ 15 ^,^ 28 ^)^. 

The items with high proportions of incorrect answers were “The IUD has many unpleasant side effects” and “The IUD increases the risk of uterine cancer”. Similar results were obtained elsewhere^(^ 21 ^)^, from data from a national survey in the United States and verified that women associated IUD with infertility and cancer. Likewise, other authors^(^ 22 ^)^ found that fear of side effects, of pain and of the infection's possibility were reasons for not using the IUD. In fact, there are two side effects commonly associated with the copper IUD, that is the only LARC available at PHCF in the SUS: increased menstrual flow and worse menstrual cramps. Other side effects are described in more specific cases and are not reported by most users of copper IUD. Very rare complications related to copper IUD are pelvic inflammatory disease and perforation of the uterine wall. No type of cancer has been associated with its use^(^ 16 ^)^.

In addition to the fear of side effects and risk of cancer, the literature also shows that women do not choose the IUD as a contraceptive method because they are afraid that the IUD might move around the body; because they are afraid of the insertion pain; because they depend on a health professional for insertion and removal of the device; because they think that there is an increased risk of ectopic pregnancy and infections; because they think that it is less effective than the pill; among other reasons^(^ 10 ^-^ 11 ^,^ 21 ^-^ 23 ^)^. 

The scenario described, in which many users do not know whether the IUD is associated with uterine cancer and what are its side effects, or mistakenly believe that it is inserted through surgery, seems to reveal that women in these three capitals would not choose a method surrounded by so many uncertainties, including doubts regarding its effectiveness and effects on the body. This is mainly because knowledge about the method might be related to its use^(^ 29 ^)^, even though this was not observed in other methods users such as emergency contraception^(^ 30 ^)^. 

In our study, we did not analyze the sources of information on the IUD, but it can be assumed that younger women access the internet to better understand the method and solve their doubts. This, on the one hand, is positive, because of the easy access to information; however, it should be noted that not all websites on contraception are reliable. A US study found that the quality of information on the IUD available on specialized websites is inconsistent and that about half of these websites provided misleading information, which could contribute to the method underutilization^(^ 31 ^)^. On the other hand, there is evidence that educational interventions increase the proportion of women with positive attitude in relation to the IUD and that women with prior knowledge around the method are more interested in using it^(^ 22 ^,^ 24 ^)^. 

Despite the low percentage of current and/or previous use of the IUD, the proportion found among the women studied was higher than the one found in the 2006 DHS^(^ 4 ^)^. However, this comparison should be carefully taken, given that women who used irreversible methods were excluded from our sample, as were those who reported not being aware of IUD, which may have overestimated the percentage. 

As observed in our findings, the level of knowledge on IUD is strongly associated with its use and interest in using it, which may be related to the fact that it is a stigmatized method, both among women and health professionals. A study^(^ 11 ^)^ conducted with US adolescents found that most did not consider the IUD as a suitable method for them. That is, adolescents do not include the IUD in the list of contraceptive methods indicated for their age group. However, the WHO considers the IUD a safe method for most women, including nulliparous women and adolescents^(^ 16 ^)^. Thus, this result may be related to the barriers imposed by health services, which mistakenly establish the minimum age of 18 years as a criterion for the availability of the IUD^(^ 7 ^)^, causing misconceptions among women who could be possible users of the method and among health professionals.

In addition to the fact that many women do not even consider the use of IUD for the reasons already mentioned, health services also have barriers imposed by outdated information regarding the criteria for its indication. An example was observed in cities in Minas Gerais, in which complementary exams, such as blood count or ultrasound, were required before the insertion of the IUD. This type of measure is not included in the protocols of the Brazilian Ministry of Health^(^ 32 ^)^.

This is a challenging context, and women’s knowledge on the IUD is only part of the challenges for up-scaling its use, which is the intention of the Brazilian Ministry of Health, as stated in an initiative launched on March 8, 2017 establishing the goal of IUD use by 10% of Brazilian women^(^ 33 ^)^.

As shown in international literature^(^ 34 ^-^ 36 ^)^, a little less than half of the women interviewed would like to use the IUD. This proportion is much higher than that observed in a study in the United States, which found 31.7% of women interested in using IUD^(^ 13 ^)^. High level of knowledge about the method was also associated with the interest in using it, as already discussed. However, among women who had no interest in using the IUD, the main reason was that they were satisfied with their current method. This finding differs from a previous study^(^ 37 ^)^, in which doubts about the effectiveness of the IUD, increased menstrual flow, pain, the possibility of acquiring infections, infertility and cancer, and the fact of having a foreign object inside the body, were the main reasons given by women for not using the IUD. 

It is interesting to note that young women, between 18 and 24 years old, showed more interest in using IUD compared to older women. It is possible that the use of a long-term method is in line with their reproductive intention to delay pregnancy. However, in the population studied, reproductive intention was not associated with interest in using IUD, but parity was. Women with children were more likely to be interested in using IUD when compared to women without children. Women in stable unions were less likely to be interested in using the method. These associations seem complex, but the profile of women who would be interested in using an IUD is clear: young, single, and with children. These women do not yet meet the requirements for adopting an irreversible method but may also not be willing to use a method that requires self-discipline and daily use, such as the contraceptive pill, making the IUD a very convenient option.

On the “CHOICE” project^(^ 38 ^)^, which offered counseling and contraceptive supplies to women at a clinic in the United States, with the advantage that the methods were available on the same day at no cost, the researchers noted that adolescents and young women, including nulliparous women, often opted for the implant or the IUD, reinforcing the fact that knowledge acquired in contraceptive counseling can make difference in the choice to use the IUD. 

Therefore, the role of health services in the expansion of IUD offer and the creation of a more favorable environment for IUD use is clear. It is precisely in primary health care services that women and couples can have more information on the availability of the method and its safety and effectiveness. The need for precise information bumps the fact that, in general, health professionals prioritize informing women and couples about how to use the method and the insertion procedures^(^ 39 ^)^, which does not always affects the decision making process. In addition, a study conducted in Minas Gerais showed that physicians are the only professionals responsible for the insertion of the IUD in that region^(^ 7 ^)^. However, it is necessary to highlight that trained and qualified nurses can insert and remove the copper IUD^(^ 1 ^)^, as they have legal competence for this practice in Brazil^(^ 40 ^-^ 41 ^)^. The inclusion of the nurse in the promotion, availability and insertion of copper IUD in PHCF can facilitate access to this method among women users of the SUS. 

Even though women from the three capitals are different from each other regarding most of the socio-demographic and reproductive characteristics investigated, there was no statistically significant difference in the interest in using IUD between women from different cities. However, women interviewed in Aracaju/SE had a higher level of knowledge about IUD than those from São Paulo/SP, even though current/previous use was similar. This leads to the conclusion that the challenge of establishing strategies that minimize the widely expressed fears and misconceptions about the IUD is not confined to one locality or region of the country.

This study has some limitations. One is that it was not possible to conduct random sampling in the second stage of the sampling process. This may have influenced the estimation of some parameters, given that, in general, women attending PHCF are those who already have children or are pregnant. However, the profile of our sample was quite diverse, including women from younger ages to older women at the end of the reproductive cycle, as well as nulliparous and women who had children. Another limitation concerns the use of the latent variable “knowledge on the IUD” and the elaboration of questions to measure the level of knowledge. Certainly, there may be divergences in the answers considered correct. For example, in the statement that the IUD is inserted through surgery, a more cautious reader might argue that the IUD may be inserted after a cesarean section, which could have confounded women participating in the study. However, we emphasize that this practice is was not common in the country by the time we interviewed women and national and international clinical guidelines emphasize that this is a simple procedure. This possible bias was minimized with a robust methodological framework, which included the use of the same questions used in various other national and international studies, the possibility of answering “I don’t know”, and the use of specific statistical tests that allowed identifying which items should compose the latent variable. In any case, we do not recommend that the adoption of the questions that made up the latent variable as a scale to be used in other studies. 

## Conclusion

This study analyzed the knowledge on the IUD and interest in using it among women of reproductive age, users of PHCF in the cities of São Paulo, Aracaju and Cuiabá, Brazil. The findings showed that the level of knowledge on IUD was higher among women who were between 25 and 34 years old, who had a higher level of education, were white, and who currently used or had used an IUD. In turn, interest in using IUD was higher among younger women, single and with children. The level of knowledge on the IUD was associated with interest in using it. Regarding the use of the IUD itself, the results confirmed that it is not frequent.
